# Leifsonia Species Bacteremia in a Hemodialysis Patient: A Difficult-to-Identify Organism

**DOI:** 10.7759/cureus.17994

**Published:** 2021-09-15

**Authors:** Mais Al-Sardi, Hiba Radwan, Ahmad B Itbaileh, Zainab AlMusa

**Affiliations:** 1 Internal Medicine, King Fahad Specialist Hospital, Dammam, SAU; 2 Internal Medicine/Infectious Diseases, King Fahad Specialist Hospital, Dammam, SAU; 3 Microbiology, King Fahad Specialist Hospital, Dammam, SAU

**Keywords:** leifsonia, bacteremia, gram positive bacilli, hemodialysis

## Abstract

*Leifsonia *is an environmental gram-positive rods bacteria. Infections due to *Leifsonia *are not common. In this report, we present a case of a hemodialysis patient with *Leifsonia* bacteremia.

A 56-year-old lady had been receiving hemodialysis through the femoral line. She presented with nonspecific symptoms. Multiple blood cultures taken from the central line and peripherally grew gram-positive bacilli, which were identified by polymerase chain reaction (PCR) as* Leifsonia *species. This serious infection resolved only after the removal of the central venous catheter (CVC) and treatment with vancomycin for four weeks from the first negative blood culture.

*Leifsonia* species are a rare cause of CVC-associated infections. *Leifsonia *should be considered in hemodialysis patients with gram-positive rod bacteremia. *Leifsonia* also has the ability to produce a biofilm. Removal of the line along with antibiotics is necessary to cure the infection.

## Introduction

*Leifsonia aquatica (L. aquatica) *is an environmental organism associated with freshwater. It was previously known as *Corynebacterium aquaticum* and was reclassified as *L. aquatica *in the year 2000. The pathogenicity of this species has not been established because of the difficulties in identification and confusion with *Aureobacterium* in previous reports. Hence, only a few case reports have been published on *L. aquatica*. It has been reported to cause septicemia in immunocompromised hosts, peritonitis in patients on continuous ambulatory peritoneal dialysis (CAPD), and bacteremia in hemodialysis patients [1,2,3]. We present a case of a tunneled femoral central venous catheter (CVC)-associated bacteremia in a hemodialysis patient due to *Leifsonia* species.

This report involves one of the rare cases in which *Leifsonia* (mainly *aquatica*), an environmental coryneform bacterium, is identified to cause infection in a hemodialysis patient. Patients on hemodialysis are at a unique risk of acquiring infections from such organisms due to immune system dysfunction and invasive devices [1].

## Case presentation

The patient was a 56-year-old female with a history of type two diabetes mellitus, atrial fibrillation, mild aortic stenosis, breast cancer treated by resection and radiotherapy, and end-stage renal disease. She had undergone two failed renal transplants. For the last six years, she had received hemodialysis as renal replacement therapy. She had been receiving hemodialysis three times per week through a femoral line that had been inserted one year prior to her current presentation. Of note, her past medical history also included gram-positive rod bacteremia related to tunneled CVC infection two years prior. The causative organism turned out to be a* Bacillus *species.

The patient presented to the emergency department with subjective fever, chills, and fatigue with no other focal symptoms. She was hypotensive with a blood pressure of 81/39 mm Hg, but other vital signs were within normal limits (temperature: 36.9° C, pulse rate: 95 beats/minute, oxygen saturation: 97% on room air). Cardiac examination showed normal first and second heart sounds, and an ejection systolic murmur grade of 3/6 was heard most loudly in the aortic area and radiated to the carotids. There was no evidence of an exit site or tunnel infection at the site of CVC, and there were no peripheral stigmata of infective endocarditis.

**Figure 1:**
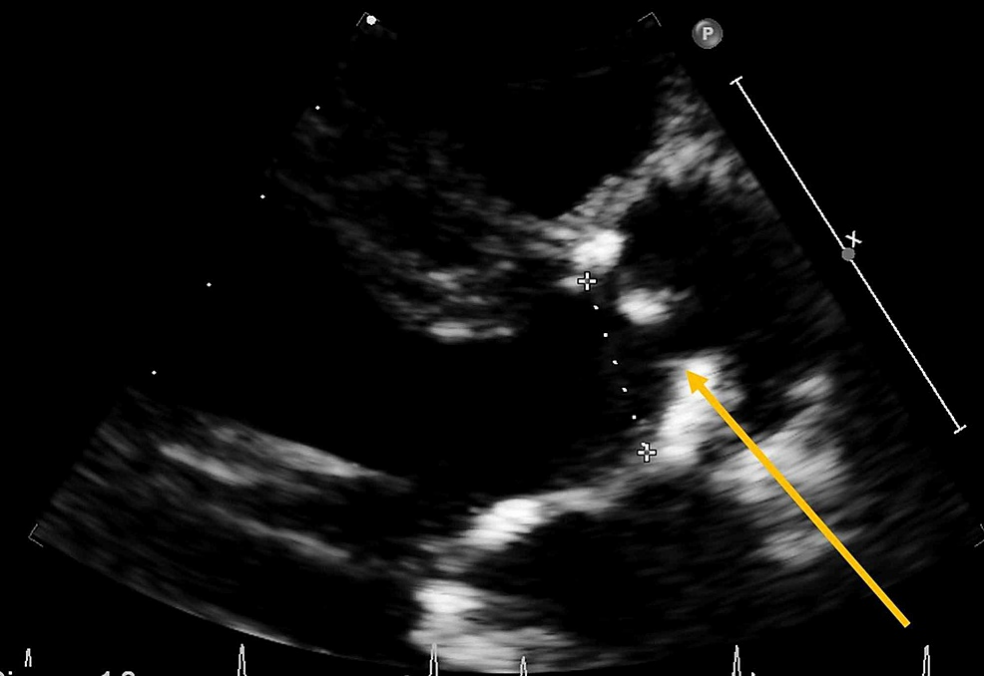
Transthoracic echocardiogram Parasternal long-axis view showing severe aortic valve stenosis

Laboratory studies showed a white cell count of 5,360 per microliter, hemoglobin level of 12 g/dL, and platelet count of 199,000 per microliter. Other basic workups, including liver and renal function tests, were within her baseline. Blood cultures were taken (central and peripheral samples), and the patient was started empirically on vancomycin and amikacin. Later, the aerobic bottles of both central and peripheral blood cultures taken initially turned out to be positive for gram-positive rods. Amoxicillin was added to cover the possibility of *Listeria* infection. A good-quality transthoracic echocardiogram was performed and showed severe aortic stenosis and no evidence of vegetation (Figure 1).

All seven blood cultures taken over a 10-day period were positive for gram-positive rods. The bacteria grew as yellow-pigmented colonies within 48 hours of incubation at 35-37 °C under 5% CO_2_. The isolate was oxidase- and catalase-positive. The isolate was loaded on a Vitek2 ANC card (BioMérieux SA, Marcy-l'Étoile, France) according to the manufacturer’s protocol, but Vitek2 failed to provide any identification (software version 7.01). The isolate was then tested in triplicate with Vitek MS matrix-assisted laser desorption/ionization time-of-flight Mass Spectrometry (MALDI-TOF) (BioMérieux) as per the manufacturer’s protocol, but the Vitek MS also could not identify the organism, and hence the sample was sent to a reference laboratory for identification (Mayo Clinic Laboratories, Rochester, MN); the organism was identified by 16S polymerase chain reaction (PCR) as belonging to *Leifsonia *species*.* Table 1 shows the susceptibility level of the organism using E-tests. It was interpreted according to the Clinical and Laboratory Standards Institute (CLSI) guidelines, which have it under related coryneform genera. Based on the final identification and biogram, amoxicillin and amikacin were stopped.

**Table 1:** Antibiotic susceptibility of Leifsonia sp. in the patient MIC: minimum inhibitory concentration

Antibiotic	MIC	Susceptibility
Cefotaxime	0.6	Sensitive
Ciprofloxacin	3	Intermediate
Penicillin G	0.38	Intermediate
Trimethoprim/sulfamethoxazole	0.023	Sensitive
Vancomycin	1	Sensitive

There was a delay in removing the line despite the persistent bacteremia, as the patient had very difficult vascular access with superior vena cava stenosis due to multiple catheter insertions. In addition, previous trials of angioplasty had failed. Eventually, the femoral line was removed, and a new right femoral line was inserted three days later. A negative blood culture at 72 hours of incubation was confirmed before inserting the new line. All blood cultures repeated after removing the line were negative. The patient improved clinically as her symptoms resolved and her blood pressure stabilized. She was discharged with the advice to continue on vancomycin intravenously for four weeks from the first negative blood culture and to maintain a vancomycin trough level between 15-20. The possibility of infectious endocarditis could not be excluded by transthoracic echocardiogram. However, we did not opt for a transesophageal echocardiogram as it is an invasive procedure, and we decided to treat her with a long course of vancomycin. The patient was seen in the infectious diseases clinic three weeks after completing the antibiotic course. She reported feeling well and had no symptoms suggestive of infection. Two sets of peripheral blood cultures were taken at that time, which did not grow any bacteria. The hemodialysis center, where the patient received dialysis sessions, had no other cases of* Leifsonia* infection or gram-positive rod bacteremia. Raising awareness among the hemodialysis unit personnel was conducted, and they were informed that this organism could be transmitted by contaminated dialysate water. We advised the personnel to report any further cases.

## Discussion

Members of the genus *Leifsonia* (formerly part of the genus *Corynebacterium*) are gram-positive aerobic rods with motile strains. They are non-acid fast, non-spore-forming, and catalase- and oxidase-positive bacteria. They grow as opaque colonies and produce a yellow pigment after extended incubation [2,4]. *Leifsonia* grows on routine microbiology media such as sheep blood agar and chocolate agar plates at 35-37 °C under 5% CO_2_, but the colonies take 48-72 hours to mature. These are usually dismissed as contaminants in clinical specimens based on their gram stain morphology unless they are isolated repeatedly from sterile specimens, “mainly blood”, in immunocompromised patients. Current commercial conventional identification systems fail to reliably identify or speciate *Leifsonia *species. Sequencing of 16S ribosomal RNA is currently the only method that can identify and speciate *Leifsonia* species with the exception of *L. aquatica*, which has also been successfully identified using MALDI-TOF technology [2]. In our case, MALDI-TOF failed to identify the organism, which makes it unlikely to be *L. aquatica*. In the literature, *L. aquatica *is the only medically relevant species. However, we reported a catheter-associated bloodstream infection caused by a *Leifsonia* species other than *aquatica.*

Patients with end-stage renal disease on hemodialysis have impaired humoral and cell-mediated immunity [1]. Therefore, these patients are at increased risk of CVC-associated infections. Gram-positive rods are not a common cause of CVC-associated infections. Our patient had experienced two different episodes of gram-positive rod bacteremia related to her dialysis access. One episode had occurred in 2016 due to* Bacillus* species, and the other episode in 2018 due to *Leifsonia*. Both episodes of bacteremia had been prolonged with multiple positive blood cultures peripherally and from the central line. In both events, removal of the line had been required to clear the bacteremia. *Leifsonia* per se is rarely a cause of such infections. *Leifsonia* has been reported as a cause of CVC-related infections in hemodialysis patients in only 10 cases [1,2,3]. We could not find any case of infective endocarditis caused by *Leifsonia* species in the literature. We felt that the high-quality transthoracic echocardiogram was an acceptable modality to make the diagnosis that infective endocarditis was less likely in our patient. Table 2 summarizes the reported cases with clinical manifestations related to *L. aquatica.*

*L. aquatica* was formerly known as *Corynebacterium aquaticum.* However, due to the chemotaxonomic and genetic differences, it was later reclassified [2,4]. *L. aquatica* was originally isolated from distilled water samples in 1962 by Leifson and continues to be a water-associated organism [5,6]. *L. aquatica*-associated infections in hemodialysis patients could be associated with bacteria contaminating dialysate water. Although dialysate water is treated with filters, deionizers, and reverse osmosis, post-treatment bacterial contamination of the dialysate water has been documented [1]. *L. aquatica* can grow under a wide range of environmental conditions, such as low temperatures and alkaline pH, and might be able to pass through polycarbonate water filters [2]. Another potential source of *Leifsonia* infection may be the oral cavity, from which species of this genus have been recently isolated [7]. *L. aquatica* has a slow growth rate and the ability to produce a biofilm, which contributes to its pathogenicity in immunocompromised patients with prosthetic devices [1]. In a study of prosthetic hip joints, *L. aquatica* was the most prevalent species identified in the biofilm of infected joints, which suggests that it may find protection in the biofilm microenvironment, especially if antibiotic courses are short [8]. This biofilm could explain why our patient’s blood culture became negative only after removing the CVC; removal of the CVC was also effective in the other reported cases with CVC-associated *L. aquatica* bacteremia [1,3]. Regarding antibiotic therapy, the optimum treatment method is still a matter of debate, but the most common antibiotic used is vancomycin while targeting a high trough level for a long course (three to four weeks) [1,2]. Since the organism isolated from our patient’s culture showed susceptibility to vancomycin, this antibiotic was the treatment given, and the patient had a good response to the vancomycin course.

**Table 2:** Summary of the reports of clinical manifestations related to Leifsonia aquatica

Infection	Number of cases	Reference
Bacteremia: patients on hemodialysis	10	[1,2,3]
Bacteremia: after retinal detachment surgery	1	[9]
Bacteremia: patient on total parental nutrition	1	[4]
Bacteremia: patient with chronic myeloid leukemia	1	[10]
Peritonitis: in peritoneal dialysis patients	2	[11,12]
Infected prosthetic hip joints: (46 bacterial isolates by 16S rRNA gene sequencing for only 10 prosthetic joints) as detailed below		[8]
Leifsonia aquatica	20	
Leifsonia shinshuensis	5	

## Conclusions

*Leifsonia species* are a rare cause of CVC-associated infections. Leifsonia should be considered in hemodialysis patients with gram-positive rod bacteremia. *Leifsonia *is an aquatic organism, and infection in hemodialysis patients may be explained by contaminated dialysate water. *Leifsonia *also has the ability to produce a biofilm. Removal of the line along with antibiotics is necessary to cure the infection.
